# Are outcomes after total knee arthroplasty worsening over time? A time-trends study of activity limitation and pain outcomes

**DOI:** 10.1186/1471-2474-15-440

**Published:** 2014-12-17

**Authors:** Jasvinder A Singh, David G Lewallen

**Affiliations:** Medicine Service, Birmingham VA Medical Center, Birmingham, AL England; Department of Medicine at School of Medicine, and Division of Epidemiology at School of Public Health, University of Alabama, Birmingham, AL England; Department of Orthopedic Surgery, Mayo Clinic College of Medicine, Rochester, MN USA

**Keywords:** Total knee replacement, Time trends, Arthroplasty, Joint replacement, Pain, Function

## Abstract

**Background:**

To examine whether function and pain outcomes of patients undergoing primary total knee arthroplasty (TKA) are changing over time.

**Methods:**

The Mayo Clinic Total Joint Registry provided data for time-trends in preoperative and 2-year post-operative activity limitation and pain in primary TKA patients from 1993-2005. We used chi-square test and analysis for variance, as appropriate. Multivariable-adjusted analyses were done using logistic regression.

**Results:**

In a cohort of 7,229 patients who underwent primary TKA during 1993-2005, mean age was 68.4 years (standard deviation (SD), 9.8), mean BMI was 31.1 (SD, 6.0) and 55% were women. Crude estimates showed that preoperative moderate-severe overall limitation were seen in 7.3% fewer patients and preoperative moderate-severe pain in 2.7% more patients in 2002-05, compared to 1992-95 (p < 0.001 for both). At 2-years, crude estimates indicated that compared to 1992-95, moderate-severe post-TKA overall limitation was seen in 4.7% more patients and moderate-severe post-TKA pain in 3.6% more patients in 2002-05, both statistically significant (p ≤ 0.018) and clinically meaningful. In multivariable-adjusted analyses that adjusted for age, sex, anxiety, depression, Deyo-Charlson index, body mass index and preoperative pain/limitation, patients had worse outcomes 2-year post-TKA in 2002-2005 compared to 1993-95 with an odds ratio (95% confidence interval (CI); p-value) of 1.34 (95% CI: 1.02, 1.76, p = 0.037) for moderate-severe activity limitation and 1.79 (95% CI: 1.17, 2.75, p = 0.007) for moderate-severe pain.

**Conclusion:**

Patient-reported function and pain outcomes after primary TKA have worsened over the study period 1993-95 to 2002-05. This time-trend is independent of changes in preoperative pain/limitation and certain patient characteristics.

**Electronic supplementary material:**

The online version of this article (doi:10.1186/1471-2474-15-440) contains supplementary material, which is available to authorized users.

## Background

Total knee arthroplasty (TKA) is an extremely effective surgical treatment option for patients with end-stage refractory knee pain and associated functional disability. National Inpatient Sample (NIS) reported that 719,000 primary TKAs were performed in the U.S. in 2010 [1], consistent with the projected estimates from a population-based study from the Olmsted County, Minnesota [2]. The estimated cost per TKA is approximately US $24,200 [3], leading to an annual cost burden of 17.6 billion for TKA in the U.S in 2010. TKA volume has increased dramatically in the last few decades [2, 4] and is projected to reach 3.5 million TKAs annually by 2030 [5].

Many studies have focused on time-trends in utilization and specific in-hospital or immediate post-arthroplasty outcomes [6–9]. To our knowledge, there are no studies assessing whether there are any appreciable time-trends in post-operative PROs, i.e., if the PROs after TKA have improved, worsened or remained stable over time. This is a critical knowledge gap since TKA is an elective surgery that results in an impressive improvement in patient-reported outcomes (PROs), including pain, function and quality of life in most patients [10]. A small but sizable proportion of patients have suboptimal pain (7-13%) and function (21-27%) outcomes post-TKA [11, 12]. Even a 1% absolute increase means an additional 7,190 patients with suboptimal outcomes annually in the U.S. Socio-demographic and clinical characteristics of patients undergoing primary TKA cohort are changing rapidly with an increasing patient complexity over time in the U.S. [13]. If there is an evolution in PRO outcomes post-arthroplasty over time, we need to know and understand that from a policy maker, clinician and patient perspective. Therefore, the objectives of this study were to: (1) examine the time trends in preoperative PROs of functional outcome and pain in patients undergoing primary TKA; (2) assess whether the risk of suboptimal PROs post-TKA has changed over time; (3) assess whether the risk of improving or worsening PROs post-TKA over time is independent of the preoperative PROs; and (4) study how these time-trends in post-TKA outcomes impact patients in different categories of age, medical and psychological comorbidity and body mass index (BMI). We hypothesized that post-TKA PROs would improve over time (hypothesis 1) and that most time-trends in improving PROs will be seen in the oldest, and in those with lower comorbidity and lower BMI (hypotheses 2-4).

## Methods

We followed the recommendations from the Strengthening of Reporting in Observational studies in Epidemiology (STROBE) statement [14] to report study methods and results.

### Study setting, data source and study eligibility

We included all patients from the Mayo Clinic Total Joint Registry who had undergone primary TKA between 1993-2005 and had completed a preoperative and/or 2-year postoperative pain and function questionnaire. The Mayo Clinic Total Joint Registry captures data on all patients who undergo knee, hip or other joint arthroplasty. For TKA, data are captured using the Mayo Knee Survey that has construct validity and reproducibility [15]. Mayo Knee Survey has questions related to pain and function, similar to the validated American Knee Society Scale [16], the most commonly used instrument in knee arthroplasty studies [17]. Several previous studies using the Mayo Knee Survey have been reported [12, 18–20]. Mayo Clinic’s Institutional Review Board approved this study.

### Predictor of interest

The main predictor of interest was time-period, divided into 1993-1995, 1996-1998, 1999-2001 and 2002-2005. Given that our objective was to assess whether the pain and activity limitation had changed over time, we compared the prevalence of moderate-severe activity limitation and moderate-severe pain in the first period, i.e., 1993-95 to the last period, i.e., 2002-05. Since the entire study period (13 years) was not an exact multiple of 3, the last period consisted of 4-years.

### Outcomes of interest

The main outcomes of interest were PROs, moderate-severe limitation of activities of daily living (ADL) and moderate-severe pain. For each of the 3 ADLs, walking, climbing stairs and getting out of a chair, patient responses were categorized into mild, moderate or severe limitation category, as previously [12, 19, 20]. Presence of ≥2 ADLs with moderate or severe limitation was classified as overall moderate-severe activity limitation *a priori* (ref, all other categories), as previously [12, 18–21]. We categorized responses to the pain question “Do you have pain in the knee in which the joint was replaced? (Please mark only one answer.)” into reference category no/mild pain (no pain, mild pain, pain with stairs only, pain with walking and stairs) or moderate-severe pain (moderate occasional, moderate continuous or severe pain), *a priori*, as previously [18, 20, 22].

### Covariates

We included several covariates (some potential confounders) that are associated with pain/function after TKA [23–26], namely: demographics (age, sex); BMI; preoperative ADL limitation; preoperative pain; and medical and psychological comorbidity. Preoperative ADL limitation and pain were assessed by questions similar to the 2-year evaluation. Medical comorbidity was assessed using validated Deyo-Charlson index [27], a weighted scale of 17 comorbidities (including cardiac, pulmonary, renal, hepatic disease, diabetes, cancer, HIV and so on), expressed as a summative score where a higher score indicates more comorbidity. We examined psychological comorbidity by the presence of anxiety and depression at the time of surgery, based on the presence of International Classification of Diseases, ninth revision (ICD-9) codes in the Mayo Clinic electronic databases.

### Bias and sample size

We included several known confounders of function and pain outcomes to reduce confounding bias, but the cohort study design raises the possibility of residual confounding bias. We accounted for non-response bias, an expected limitation of this study, by presenting the non-responder characteristics and considering them in study interpretation. We did not perform any formal sample size calculations, since we anticipated >6,000 patients at both baseline and 2-year follow-up for our study, to provide adequate number of outcomes. Correlation of observations (bilateral TKA in a patient, simultaneous or sequential) was accounted for by using appropriate statistical methods.

### Statistical analyses

We compared crude rates of moderate-severe ADL limitation and pain across the four pre-specified time periods using chi-square tests. We used univariate and multivariable-adjusted logistic regression models, using a generalized estimating equations (GEE) approach, to assess the association between the most recent time period (i.e., 2002-05 compared to 1992-95) and moderate-severe ADL limitation/pain 2-years after primary TKA. This method adjusted for the correlation between observations on the same subject due to both knees having been replaced. As specified *a priori*, we compared the first and the last time-periods. The multivariable models were hierarchical and included the variables: (1) model 1: age and sex; (2) model 2: model 1+ medical comorbidity; (3) model 3: model 2 + anxiety + depression; (4) model 4: model 3 + medical comorbidity; (5) model 5: model 4 + preoperative pain/ADL limitation (in the respective model); (5) model 6: model 5 + BMI.

Subgroup analyses were done by examining multivariable-adjusted model 5 in patient subpopulations of: (1) age, <65 vs. ≥65; (2) psychological morbidity, defined as presence of anxiety and/or depression (none vs. any); (3) BMI, <30 vs. ≥30; and (4) Deyo-Charlson index (zero vs. one or higher). Odds ratios (OR), 95% confidence intervals (CI) and p-values were calculated. A p-value <0.05 was considered statistically significant.

## Results

### Study cohort characteristics and crude rates of preoperative and 2-year postoperative functional limitation and pain

7,229 patients underwent primary TKA at our institution 1993-2005 and completed baseline preoperative assessments and 7,139 patients who completed 2-year assessments (Table 1). For the preoperative cohort, 55% were women and 81% were older than 60 years. The mean (standard deviation) age, BMI and Deyo-Charlson comorbidity index for the study cohort were 68.4 (9.8), 31.1 (6.0) and 1.3 (2.0), respectively. Osteoarthritis was the underlying diagnosis in 94%. Characteristics of 2-year cohort were similar to the pre-operative cohort (Table 1).

**Table 1:** **Characteristics of the primary TKA cohort preoperatively and at 2**-**year follow**-**up**

	Preoperatively (n = 7,229)	2-year (n = 7,139)
**Gender**		
**Females**	55%	56%
**Males**	45%	44%
**Age**		
**≤60**	19%	18%
**>60-** **70**	35%	35%
**>70-** **80**	38%	38%
**>80**	8%	8%
**BMI**		
**<25**	13%	13%
**25.1-** **30**	35%	35%
**30.1-** **35**	30%	29%
**35.1-** **40**	14%	14%
**>40**	8%	9%
**ASA class**		
**I**-**II**	57%	58%
**III**-**IV**	43%	42%
**Diagnosis**		
**Inflammatory arthritis**	4%	4%
**Osteoarthritis**	94%	94%
**Other**	2%	2%
**Cement fixation**		
**Uncemented**	2%	2%
**Cemented/** **hybrid**	98%	98%

BMI, Body mass index.

31 missing for BMI, 30 missing for ASA score.

#### Preoperative

Preoperative limitations in walking, climbing stairs and rising from chair showed significant time-trends (p ≤ 0.007), with a lower proportion of patients with moderate or severe activity limitations in the more recent period compared to the earlier time-period, except for stair climbing (Table 2). Additional file 1 provides detailed assessment of limitations for ADLs and pain over time. Preoperative dependence on walking aids decreased significantly over time (p = 0.025). Overall moderate-severe functional limitation, defined as two or more ADLs with moderate or severe limitations, were noted in 75.2%, 72.1%, 65.4% and 67.9% patients in the four time-periods, indicating a statistically significant decline in preoperative ADL limitations over time (p < 0.001; Table 2). The reduction in overall moderate-severe preoperative ADL limitation was attributable mainly to reduction in limitation in getting out of chair (18.4% fewer) and walking (5.7% fewer). The proportion of patients with moderate/severe preoperative pain increased from 64.3% in 1993-95 to 67% in 2002-05 with a significant time-trend (p < 0.001; Table 2).

**Table 2:** **Time**-**trends in moderate**-**severe preoperative pain and functional limitation**

	1993-1995 (n ~ 1,510)*	1996-1998 (n ~ 1,646)*	1999-2001 (n ~ 1539)*	2002-2005 (n ~ 2,480)*	Absolute Difference (period 1 -period 4)	p-value for time-trend
Preoperative walking limitations						<0.001
None/Mild	25.7%	25.9%	32.2%	31.4%		
Moderate/Severe	74.3%	74.1%	67.8%	68.6%	-5.7%	
Preoperative stairs limitations						0.007
None/Mild	14.4%	15.5%	16.7%	12.7%		
Moderate/Severe	85.6%	84.5%	83.3%	87.3%	+1.7%	
Preoperative chair limitations						<0.001
None/Mild	58.4%	65.3%	71.2%	76.8%		
Moderate/Severe	41.6%	34.7%	28.8%	23.2%	-18.4%	
Preoperative walking aids						0.025
No/mild dependence	70.9%	71.9%	72.0%	75.3%		
Moderate dependence	18.3%	16.4%	16.7%	15.4%	-2.9%	
Complete dependence or unable to walk	10.8%	11.7%	11.3%	9.3%	-1.5%	
Preoperative overall ADL limitations						<0.001
None/Mild	24.8%	27.9%	34.6%	32.1%		
Moderate/Severe	75.2%	72.1%	65.4%	67.9%	-7.3%	
Preoperative pain						<0.001
None/Mild/Stairs/Stairs and walking	35.7%	39.4%	41.8%	33.0%		
Moderate/Severe	64.3%	60.6%	58.2%	67.0%	+2.7%	

ADL, activities of daily living.

*Missingness ranged from 1% to 3% for various variables, since not all the patients answered each question on the survey.

#### Postoperative

Compared to 1992-95, moderate-severe post-TKA overall ADL limitation was seen in 4.7% more patients and moderate-severe post-TKA pain in 3.6% more patients in 2002-05, both statistically significant (p ≤ 0.018; Table 3). Compared to 1992-95, 8.6% and 5.9% more patients had moderate or severe limitations in walking and stairs post-TKA during 2002-05, and 1.3% fewer had moderate-severe limitations in getting out of chair (Table 3). The proportion of patients with moderate/severe post-TKA pain increased from 5.0% in 1993-95 to 8.6% in 2002-05 with a significant time-trend (p < 0.001; Table 2), an almost doubling of the incidence.

**Table 3:** **Time**-**trends in 2**-**year post**-**primary TKA pain and functional limitation outcomes**

	1993-1995 (n ~ 1,510)*	1996-1998 (n ~ 1,646)*	1999-2001 (n ~ 1539)*	2002-2005 (n ~ 2,480)*	Absolute Difference (period 1 -period 4)	p-value for time-trend
2-year walking limitations						<0.001
None/Mild	80.4%	75.0%	72.9%	71.8%		
Moderate/Severe	19.6%	25.0%	27.1%	28.2%	+8.6%	
						0.001
2-year stairs limitations						
None/Mild	62.6%	59.5%	62.0%	56.6%		
Moderate/Severe	37.4%	40.5%	38.0%	43.3%	+5.9%	
2-year chair limitations						0.57
None/Mild	91.0%	91.7%	92.1%	92.3%		
Moderate/Severe	9.0%	8.3%	7.9%	7.7%	-1.3%	
2-year walking aids						0.14
No/mild dependence**	88.0%	88.3%	88.9%	88.8%		
Moderate dependence	4.5%	5.8%	5.4%	4.9%	+0.4%	
Complete dependence or unable to walk	7.5%	5.9%	5.7%	6.2%	-0.7%	
2-year overall ADL limitations						0.018
None/Mild	81.0%	77.3%	77.7%	76.3%		
Moderate/Severe	19.0%	22.7%	22.3%	23.7%	+4.7%	
2-year TKA pain						<0.001
None/Mild/Stairs/Stairs and walking	95.0%	93.3%	93.5%	91.4%		
Moderate/Severe	5.0%	6.7%	6.5%	8.6%	+3.6%	

*Missingness ranged from 1% to 3% for various variables, since not all the patients answered each question on the survey.

**No/mild dependence, no supports or cane long walk.

### Did post-TKA overall moderate-severe ADL limitation and moderate-severe pain decrease over time? (Hypothesis 1)

Unadjusted analyses showed that the odds of moderate-severe ADL limitation and moderate-severe pain at 2-year postoperative were significantly higher in 2002-05 compared to 1992-95 (Table 4). Multivariable hierarchical models showed that odds were minimally attenuated after adjustment, contrary to our hypothesis that PROs would improve over time (Table 4). Adjustment for preoperative pain/limitation, arguably the most important variable, led to higher odds ratios of 1.34-1.40 for moderate-severe ADL limitation and 1.79-1.80 for moderate-severe pain.

**Table 4:** **Univariate and Multivariable**-**adjusted odds of post**-**TKA moderate**-**severe ADL limitation and moderate**-**severe pain in 2002**-**05 compared to 1993**-**1995**

	Odds of outcome in 2002-05 compared to 1993-1995	
	Odds ratio (95% CI)	p-value
**2-** **year Overall Moderate**-**severe ADL limitation**		
Unadjusted	**1.32** **(1.09**, **1.61)**	**0.005**
Age, sex adjusted	**1.31** **(1.06**, **1.60)**	**0.011**
Age, sex, comorbidity adjusted	**1.24** **(1.01**, **1.52)**	**0.044**
Age, sex, anxiety, depression adjusted	1.21 (0.98, 1.49)	0.071
Age, sex, comorbidity, anxiety, depression adjusted	1.17 (0.95, 1.44)	0.146
Age, sex, comorbidity, anxiety, depression, preoperative ADL limitation adjusted	**1.40** **(1.07**, **1.83)**	**0.015**
Age, sex, comorbidity, anxiety, depression, preoperative ADL limitation, BMI adjusted	**1.34** **(1.02**, **1.76)**	**0.037**
**2-** **year Moderate**-**Severe Pain**		
Unadjusted	**1.79** **(1.30**, **2.45)**	<**0.001**
Age, sex adjusted	**1.74** **(1.26**, **2.39)**	**0.001**
Age, sex, comorbidity adjusted	**1.72** **(1.25**, **2.37)**	**0.001**
Age, sex, comorbidity, anxiety, depression adjusted	**1.65** **(1.19**, **2.28)**	**0.002**
Age, sex, comorbidity, anxiety, depression, preoperative pain adjusted	**1.80** **(1.17**, **2.75)**	**0.007**
Age, sex, comorbidity, anxiety, depression, preoperative pain, BMI adjusted	**1.79** **(1.17**, **2.75)**	**0.007**

Bold indicates significant odds ratios.

### Did time-trends in post-TKA outcomes differ by age, comorbidity and BMI? (Hypotheses 2-4)

Subgroup analyses were performed by age, psychological comorbidity, BMI and Deyo-Charlson comorbidity. The increased odds of moderate-severe ADL limitation and moderate-severe pain in 2002-05 compared to 1992-95 persisted in patients 65 years and older, but not in patients younger than 65 years (Table 5). Patients without psychological comorbidity had significantly higher odds of moderate-severe ADL limitation and moderate-severe pain in 2002-05 compared to 1992-95 (Table 5), but not patients with psychological comorbidity. The increased odds of moderate-severe pain in 2002-05 compared to 1992-95 were evident for patients with Deyo-Charlson index of 1 or higher or BMI ≥30, but not in those with Deyo-Charlson index of zero or BMI <30 (Table 5).

**Table 5:** **Subgroup Analyses of the time**-**trends in post**-**TKA moderate**-**severe ADL limitation and moderate**-**severe pain in 2002**-**05 compared to 1993**-**1995 by important patient characteristics**

	Odds ratio (95% CI)	P-value	Odds ratio (95% CI)	P-value
	**Age** **<65**		**Age** ≥**65**	
**2-** **yr Overall Moderate**-**severe ADL limitation** ^**1**^	0.70 (0.41, 6.05)	0.19	**1.43** **(1.06**, **1.95)**	**0.021**
**2-** **yr Mod-** **Severe Pain** ^**1**^	1.63 (0.77, 3.47)	0.20	**1.81** **(1.07**, **3.07)**	**0.026**
	**No Psychological morbidity**		**Psychological morbidity**	
**2-** **yr Overall Moderate-** **severe ADL limitation** ^**2**^	**1.50** **(1.12**, **2.00)**	**0.006**	0.74 (0.29, 1.93)	0.54
**2**-**yr Mod-** **Severe Pain** ^**2**^	**1.61** **(1.02**, **2.53)**	**0.039**	3.82 (0.87, 16.7)	0.076
	**BMI** **<30**		**BMI** ≥**30**	
**2-** **yr Overall Moderate-** **severe ADL limitation** ^**3**^	1.11 (0.75, 1.65)	0.60	1.36 (0.95, 1.95)	0.094
**2-** **yr Mod-** **Severe Pain** ^**3**^	1.44 (0.82, 2.53)	0.20	**2.19** **(1.11**, **4.31)**	**0.023**
	**Deyo**-**Charlson index 0**		**Deyo**-**Charlson index 1 or higher**	
**2-** **yr Overall Moderate-** **severe ADL limitation** ^**4**^	1.48 (1.00, 2.20)	0.053	1.09 (0.76, 1.56)	0.62
**2-** **yr Mod-** **Severe Pain** ^**4**^	1.56 (0.88, 2.74)	0.125	**2.16** **(1.12**, **4.15)**	**0.021**

Psychological comorbidity was defined as the presence of anxiety, depression or both.

^1^adjusted for age, sex, comorbidity, anxiety, depression, BMI, preoperative pain/function.

^2^adjusted for age, sex, comorbidity, BMI, preoperative pain/function (not adjusted for anxiety or depression, since the groups were stratified by its presence).

^3^adjusted for age, sex, comorbidity, anxiety, depression, BMI, preoperative pain/function (not adjusted for BMI, since the groups were stratified by BMI).

^4^adjusted for age, sex, anxiety, depression, BMI, preoperative pain/function (not adjusted for comorbidity, since the groups were stratified by Deyo-Charlson comorbidity score).

Bold indicates significant odds ratios.

## Discussion

We found a significant increase in the odds of suboptimal post-primary TKA function and pain outcomes from 1992-95 to 2002-05. In contrast, preoperative moderate-severe ADL limitation decreased and preoperative moderate-severe pain minimally increased from 1993-95 to 2002-05. The adjusted odds of 2-year post-TKA moderate-severe ADL limitation increased by 34% over the study period, while the odds of 2-year post-TKA moderate-severe pain increased by 79%. The absolute increases in post-TKA overall moderate-severe ADL and pain limitations between the first and last time-periods were 4.7% (19% vs. 23.7%) and 3.6% (5% vs. 8.6%), statistically significant and clinically meaningful. These findings add to the current knowledge in post-arthroplasty PROs. We are not aware of any other study of time-trends in PROs after primary TKA.

Our study addressed our first hypothesis, i.e., time-trends in post-TKA function and pain outcomes, which contrary to our expectation, actually worsened over time. Medical [25, 28, 29] and psychological comorbidity [30–32] and obesity [33], factors associated with more complications and/or worse PROs after primary TKA, have worsened over time [6, 13] in patients undergoing primary TKA. Interestingly, adjusting for preoperative medical and psychological comorbidity, age, BMI and other confounders (sex and preoperative pain and ADL limitation) had no/minimal effect on time-trends in post-TKA moderate-severe ADL limitation or pain. This implies that increasing comorbidity, BMI, preoperative pain and ADL limitation over time do not explain the time-related worsening in post-TKA ADL limitations and pain outcomes. An impressive finding was an almost doubling of moderate-severe pain from 5% to 8.5% over the 13-year study period. This increase in the proportion of patients with suboptimal pain outcomes 2-year post-primary TKA over 13-years was an unexpected finding that needs further study. This 8.5% annual rate translates into 61,115 moderate-severely painful TKAs in 2010 alone, which represents a significant public health and patient burden.

So, why in the face of lower proportion of patients with moderate-severe ADL limitation (albeit higher proportion with moderate-severe pain) preoperatively, are postoperative pain and function outcomes worsening over time? Since our final models were adjusted for preoperative status and various other important factors, the time-trends in post-operative pain and function are in fact independent of changes in preoperative pain and function, respectively. We think that the increase in odds of moderate-severe ADL limitation over time may be related to shorter inpatient stay and a lower proportion discharged to home over time [6], both of which may lead to more suboptimal recovery; on the other hand, recent improvements in pre- and post-operative rehabilitation programs might be expected to improve post-TKA functional outcomes [34]. Potential causes for worsening pain outcomes over time may be the increasing prevalence of osteoarthritis over time [35, 36], which may lead to higher likelihood of other lower extremity joint involvement or the spine, which may contribute to persistent index TKA pain due to referred pain from another osteoarthritic joint (i.e. hip or back) or due to interference with optimal physical therapy and rehabilitation post-TKA [37, 38]. The increase in odds of 1.34 in moderate-severe ADL limitation was more modest as compared to the increase in odds of moderate-severe pain (OR, 1.77).

Future studies need to examine whether other factors such as psychological distress (e.g. post-traumatic stress disorder), coping strategies, family support, pain medication use in the postoperative period and/or type of rehabilitation, can explain the worsening pain and function outcomes after primary TKA over time. We used a diagnostic code, not a questionnaire, for depression/anxiety and therefore we may have missed subclinical or undiagnosed disease. Severity of depression or anxiety was not measured in our study, which may also contribute to worsening of persistent pain/functional limitation post-TKA. Over time, TKA has been made available to a wider patient population, some of whom may have been excluded previously, due to an unfavorable patient profile, not likely to benefit optimally from TKA. Worsening in other patient characteristics (proportion with fibromyalgia, catastrophizing etc.) over time not measured in our study may also be contributing to worsening outcomes over time.

Our subgroup analyses provided important insights. We hypothesized that post-TKA pain and function outcomes would improve over time in the oldest, and in those with lower comorbidity and lower BMI. We noted worse pain and function outcomes in 2002-05 compared to 1993-95 in the patients 65 years and older and those without psychological comorbidity. This was contrary to our expectations, since we hypothesized that patients with psychological comorbidity and those in the younger age group (who also have worse psychological comorbidity) might be the ones at highest risk of worsening PRO outcomes over time. These associations were also independent of sex, preoperative pain/function, BMI and preoperative comorbidity. To our knowledge, this is the first study to report these findings. Another novel finding was that pain outcomes in the 2002-05 were worse compared to 1993-95 in patients with Deyo-Charlson index of one or more or BMI ≥30. Thus, our study identified specific patient populations who demonstrate worsening PRO outcomes post-TKA. Patients with these characteristics should be the focus of the future studies to investigate the reasons for worsening PROs over time and design strategies to improve PROs.

Study findings must be interpreted considering the study limitations. Non-response may have biased our results. Non-responders to 2-year survey were more likely than responders to have slightly higher co-morbidity, higher ASA class, greater distance to the medical center and were younger. However, the direction of bias is unclear since it’s not known how the patient characteristics associated with non-response might have impacted these time-trends. This is single center study and generalizability to other settings may be challenging. The similarity of our cohort to other published cohorts [39–41] as well as a national U.S. sample [7], supports the representativeness of our sample. Use of diagnostic codes for medical and surgical comorbidity may have led to under-recognition of these conditions, which would bias study results towards null, making our estimates conservative. Deyo-Charlson index that uses diagnostic codes is a validated comorbidity measure [27] and the prevalence of depression using codes is similar to the 9-15% reported in studies using validated instruments for depression [42–44]. The use of multi-modal pain protocols, in- and outpatient rehabilitation programs, practice of earlier joint mobilization as well as prosthesis and surgery techniques may have evolved over time and could have positively or negatively impacted pain outcomes, especially in the last few years. We could not explore these trends in the more recent as well as assess the contribution of these potential secular trends due to limited resources and lack of availability of these data in our databases. This should be explored in future studies. Our study had several strengths. We used a large sample from a Total Joint Registry, and adjusted analyses for potential confounders and covariates of pain and functional outcomes.

## Conclusion

In conclusion, we found that post-TKA PRO outcomes 2 years after primary TKA had worsened from 1993-95 to 2002-05. In particular, the odds of moderate-severe ADL limitation and pain were higher in the most recent time-period. Time-related worsening of pain and ADL outcomes was independent of pre-operative ADL limitation and pain and other factors associated with post-TKA pain and functional limitation. The increase in odds was noted in particular in patients with older age, no psychological morbidity, obesity and higher medical comorbidity. Future studies are needed to identify the reasons for worsening pain and functional outcomes in general and in these groups of patients so that interventions can be targeted for improving post-arthroplasty outcomes.

### IRB approval

The Mayo Clinic Institutional Review Board approved this study and all investigations were conducted in conformity with ethical principles of research.

## Grant support

JAS is supported by grants from the Agency for Health Quality and Research Center for Education and Research on Therapeutics (AHRQ CERTs) U19 HS021110, National Institute of Arthritis, Musculoskeletal and Skin Diseases (NIAMS) P50 AR060772 and U34 AR062891, National Institute of Aging (NIA) U01 AG018947, and National Cancer Institute (NCI) U10 CA149950, and research contract CE-1304-6631 from Patient Centered Outcomes Research Institute (PCORI). JAS is also supported by the resources and the use of facilities at the VA Medical Center at Birmingham, Alabama, USA.

## Role of the funding sources

The funding sources played no role in development of the study protocol, data analyses or interpretation, preparation of the manuscript or the decision to submit it.

## Electronic supplementary material

Additional file 1:Time trends in unadjusted rates of preoperative pain and activity limitation.(DOCX 69 KB)

## Abbreviations

TKA: Total knee arthroplasty
BMI: Body mass index
PRO: Patient-reported outcomes
ADL: Activities of daily living.
