# Supplementary material for: Are outcomes after total knee arthroplasty worsening over time? A time-trends study of activity limitation and pain outcomes
Source: BMC Musculoskelet Disord. 2014 Dec 17;15:440. doi: 10.1186/1471-2474-15-440 (PMC4301928; doi:10.1186/1471-2474-15-440)
Supplement: Supplementary file 1 — Additional file 1:Time trends in unadjusted rates of preoperative pain and activity limitation.(DOCX 69 KB) [file 12891_2014_2368_MOESM1_ESM.docx]

**Appendix**

**Appendix 1. Time trends in unadjusted rates of preoperative pain and activity limitation**

|  | 1993-1995  (n~1,510)* | 1996-1998  (n~1,646)* | 1999-2001  (n~1539)* | 2002-2005  (n~2,480)* | p-value of time-trend |
| --- | --- | --- | --- | --- | --- |
|  |  |  |  |  |  |
| Pain, % |  |  |  |  | <0.001 |
| None | 0.7 | 1.0 | 1.1 | 0.7 |  |
| Mild, occasional | 1.6 | 2.7 | 2.3 | 1.5 |  |
| Stairs only | 2.6 | 1.1 | 1.1 | 0.8 |  |
| Walking & stairs | 28.9 | 32.4 | 34.3 | 28.6 |  |
| Moderate, occasional | 23.6 | 24.5 | 23.4 | 26.6 |  |
| Moderate, continuous | 32.5 | 26.8 | 25.2 | 29.0 |  |
| Severe | 8.1 | 9.4 | 9.7 | 11.4 |  |
|  |  |  |  |  |  |
| Moderate-Severe pain, % | 65.6 | 62.0 | 60.0 | 67.9 | <0.001 |
|  |  |  |  |  |  |
| Preoperative walking limitations, % |  |  |  |  | <0.001 |
| None | 11.2 | 14.6 | 14.7 | 13.4 |  |
| Mild | 14.5 | 15.9 | 17.2 | 15.7 |  |
| Moderate | 58.5 | 55.9 | 56.6 | 57.0 |  |
| Severe | 15.7 | 13.6 | 11.5 | 13.8 |  |
|  |  |  |  |  |  |
| Preoperative stairs limitations, % |  |  |  |  | 0.01 |
| None | 3.6 | 3.2 | 3.2 | 3.2 |  |
| Mild | 10.8 | 12.3 | 13.4 | 9.5 |  |
| Moderate | 76.2 | 75.8 | 74.8 | 79.5 |  |
| Severe | 9.5 | 8.8 | 8.5 | 7.8 |  |
|  |  |  |  |  |  |
| Preoperative chair limitations, % |  |  |  |  | <0.001 |
| None | 7.2 | 10.1 | 10.9 | 9.3 |  |
| Mild | 51.3 | 55.2 | 60.2 | 67.6 |  |
| Moderate | 40.5 | 34.2 | 28.1 | 23.0 |  |
| Severe | 1.1 | 0.5 | 0.7 | 0.2 |  |
|  |  |  |  |  |  |
| Preoperative overall limitations, % |  |  |  |  |  |
| None/mild | 24.8 | 27.9 | 34.6 | 32.1 |  |
| Moderate/ Severe | 75.2 | 72.1 | 65.4 | 67.9 |  |
|  |  |  |  |  |  |
| Preoperative walking ability, % |  |  |  |  | <0.001 |
| Unlimited | 4.0 | 3.8 | 5.5 | 6.0 |  |
| >10 blocks | 7.0 | 7.4 | 9.9 | 8.2 |  |
| 5-10 blocks | 14.0 | 14.2 | 15.8 | 16.7 |  |
| <5 blocks | 57.1 | 56.2 | 52.8 | 56.5 |  |
| Housebound/ indoors only | 14.6 | 15.8 | 12.4 | 10.6 |  |
| Unable | 0.8 | 0.4 | 0.6 | 0.4 |  |
|  |  |  |  |  |  |
| Preoperative supports and walking aids, % |  |  |  |  | <0.001 |
| None | 57.9 | 58.6 | 58.4 | 61.7 |  |
| Cane long walks | 13.0 | 13.3 | 13.6 | 13.6 |  |
| Cane full time | 18.3 | 16.4 | 16.7 | 15.4 |  |
| Crutch | 1.2 | 1.6 | 1.6 | 1.0 |  |
| 2 canes | 1.3 | 1.0 | 0.4 | 0.2 |  |
| 2 crutches | 2.3 | 1.9 | 2.1 | 1.1 |  |
| Walker | 3.2 | 4.1 | 4.3 | 5.0 |  |
| Unable to walk | 0.6 | 0.6 | 0.8 | 0.4 |  |
|  |  |  |  |  |  |
| Preoperative knee braces, % |  |  |  |  | <0.001 |
| No | 93.4 | 90.2 | 89.7 | 89.8 |  |
| Yes | 4.2 | 7.2 | 7.9 | 8.2 |  |

*Missingness ranged from 1% to 3% for various variables, since not all the patients answered each question on the survey
